# ERG shrinks by 10% when reducing dark adaptation time to 10 min, but only for weak flashes

**DOI:** 10.1007/s10633-020-09751-6

**Published:** 2020-01-29

**Authors:** Michael Bach, Cornelia Meroni, Sven P. Heinrich

**Affiliations:** 1 Eye Center, Medical Center, University of Freiburg, Killianstr. 5, 79106 Freiburg, Germany; 2 Faculty of Medicine, University of Freiburg, Freiburg, Germany

**Keywords:** ISCEV ERG Standard, Dark-adapted ERG, Dark adaptation, Time

## Abstract

**Purpose:**

To compare dark-adapted (DA) ERG between 10, 15 and 20 min of dark adaptation (DA).

**Methods:**

In a counterbalanced random block design, 40 healthy adult subjects were dark-adapted for 10, 15 or 20 min before we recorded ERGs to nine flash strengths from 0.001 to 10.0 cd s/m^2^ (dilated pupils) with a DTL-like electrode. Before and between sessions, the room was lit. Apart from choosing a wider range of stimulus strengths, and adding shorter DA times, the recordings fully complied with the ISCEV ERG Standard, namely using corneal electrodes, mydriasis and a standard DA sequence.

**Results:**

The a-wave amplitude was not affected by any adaptation condition. For the b-wave amplitude, effects of reduced DA time are stronger for weaker flashes: Reducing DA from 20 to 10 min had no measurable effect on the DA 3 ERG, but reduced the DA 0.01 b-wave significantly (*p* < 0.0001) to 87 ± 2% (mean ± SEM). The DA 0.001 b-wave (not part of the ISCEV ERG Standard) was more affected (down to 72 ± 4%). There was a small, but significant, increase, only for weak flashes, in a- and b-wave peak times for 20 compared to 10-min dark adaptation time.

**Conclusion:**

Reducing dark adaptation time from 20 to 10 min in normal participants has no effect on the ISCEV DA 3 and DA 10 ERG. The reduction in DA 0.01 ERGs to 87 ± 2% agrees with Hamilton and Graham (Doc Ophthalmol 133:11–19, 2016. 10.1007/s10633-016-9554-x) who found 90 ± 2% and with Asakawa et al. (Doc Ophthalmol 139:33–44, 2019. 10.1007/s10633-019-09693-8) who found 83%. Pending verification in pathophysiological states, the current results suggest that one might be able to correct for the 10% amplitude loss when gaining 10 min through shortened DA.

## Introduction

The first ISCEV ERG Standard [1] prescribed 20 min of dark adaptation (DA) before applying the dim flashes (0.01 cd s/m^2^) to record the dark-adapted response, representing the rod system. There is no rationale given for this number in that paper, and in all likelihood, it represents a compromise.

Recently, Hamilton and Graham [2] have addressed this void and assessed the effect of reduced DA, testing a series of different durations down to 1 min. They found no sizable effect on the ISCEV DA 3.0 response, while the DA 0.01 was markedly reduced in amplitude and peak time with short adaptation durations. At intermediate conditions, only small effects were found. For instance, with 10 min of DA, the amplitude was reduced by 10% and peak times were slightly shorter. Asakawa et al. [3] have recently extended this (also using skin electrodes) to cone responses and pupillography, and for the dark-adapted responses found highly similar effects as Hamilton and Graham [2].

The present study has two aims:

1. Given the possibly important implications of these findings for future revisions of the ISCEV ERG Standard [4], the present study aims at corroborating them specifically at intermediate DA durations, while fully following the ISCEV ERG Standard with respect to pupil dilation, corneal electrodes and measuring not during, but after dark adaptation.
2. In order to better understand the characteristics of the effect, we tested how the responses to even weaker flash stimuli, below those prescribed by the ISCEV ERG Standard, are affected by shortened DA. We hypothesized that the effect of adaptation duration would be larger for weaker flashes. Furthermore, we also employed the “DA 10.0” condition as suggested in the latest ISCEV ERG Standard.

## Methods

### Participants

We included 40 healthy participants, most of them staff of the University of Freiburg Eye Center. Their age was 30 ± 3 years (mean ± SD), and they reported no ophthalmological or neurological disorder. The study was approved by the institutional review board (#582-15), and all participants provided written informed consent.

### Protocol

The study protocol followed the ISCEV ERG Standard [4] diligently, with three exceptions:

- Only the dark-adapted responses were measured.
- In addition to the dark-adapted 0.01 ERG and the dark-adapted 3 ERG, we started with a ten times weaker flash and incremented in half-log unit steps, thus using nine flash strengths: 0.001, 0.003, 0.01, 0.03, 0.1, 0.3, 1.0, 3.0 and 10.0 cd s/m ^2^.
- The dark adaptation time varied between three values: 10 (“AT10”), 15 (“AT15”) and 20 min (“AT20”). Every participant underwent all three DA times in a block-randomized fashion: For the first participant, the sequence was *abc*, for the next it was *acb*, then *bac*, etc.

Dark adaptation and recordings took place in a light-proof room. The participant area in that room had black walls and was separated from the examiner by an additional light-proof curtain. During dark adaptation and recordings, all room lights were turned off, and the computer display for the examiner was switched to red-only. All control lights were covered.

Before each period of dark adaptation, participants were in a lit room for at least 15 min. As the participants were allowed to freely look around in the room, actual pupil illumination could vary dynamically in the range of approximately 10–200 lx.

The ERG was only recorded from one eye, which was dilated using the mydriatic tropicamide. For each participant, all recordings were performed in a single session which lasted 100–120 min. Electrodes were attached before the first dark adaptation period.

### Recording

The stimulator was a Q450 from Roland Consult, driven with in-house software [5], written in Objective-C, as used, for instance, in [6]. Stimulus strength was calibrated with the Optometer P 9710 Gigahertz-Optik with the photopic sensor VL-3701-2.

**Fig. 1:**
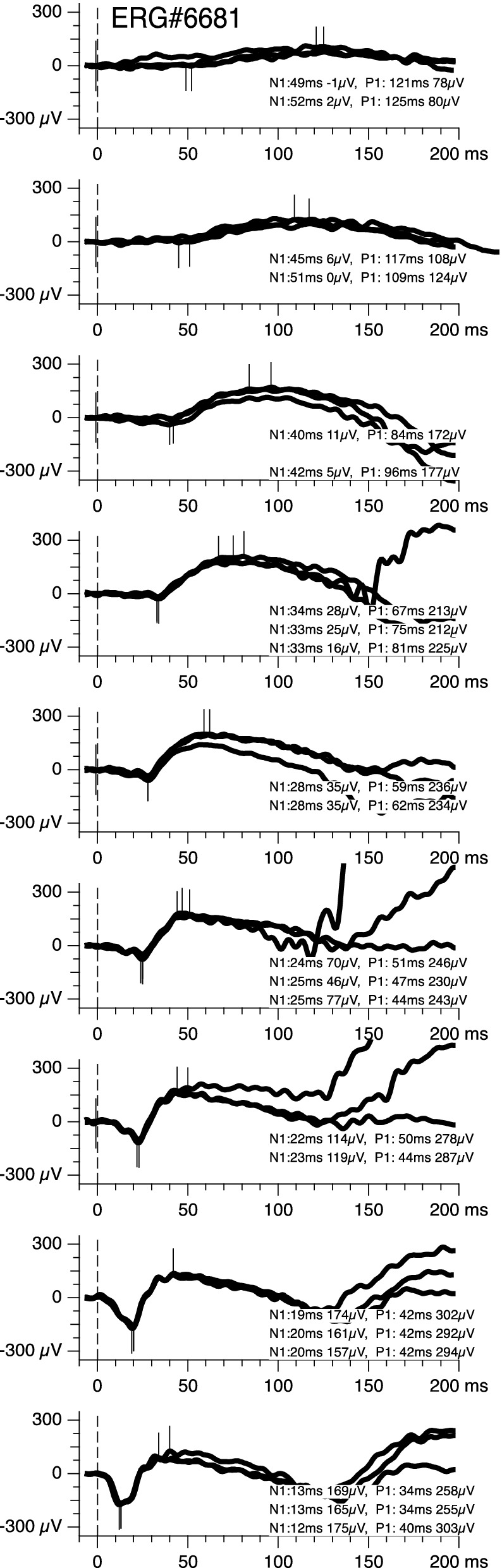
Raw ERG traces from a representative participant in this experiment. Flash strength increases from top (0.001) to bottom (10 cd s/m²). Traces represent individual takes without averaging. Peaks settings were manually adjusted; some traces were not included (e.g., fifth from top, only two traces were selected as indicated by the missing ticks at the lower one of the three takes)

Flashes were triggered manually when the operator saw a clean baseline. At least three responses were obtained for every flash strength, and no response averaging was used. One measurement run, including all flash strengths, but excluding dark adaptation time, lasted approximately 7 min. For analysis, all records were scrutinized, traces with relevant artifacts were discarded, the peak locations as suggested by the software (custom analysis scripts based on Igor Pro, Wavemetrics) adjusted where necessary (see Fig. 1 for examples), and a- and b-wave parameters were measured as recommended by the ISCEV ERG Standard [4]. The average of the a- and b-waves of the two or three traces per flash strength entered further analysis.

### Analysis

All peak measures were further analyzed with R (version 3.5 [7]). The preliminary analysis of recordings from the first few participants showed that the effects were small and monotonous, meaning the AT15 values were “between” (apart from noise) the AT10 and AT20 conditions. We still pursued the initial protocol, but did not enter the AT15 values into statistical analysis in order to conserve power. Thus, the statistical models used the factors FLASH_STRENGTH with nine levels and DA_TIME with two levels.

## Results

The peak locations (Fig. 1) were analyzed with respect to the a-wave amplitude and peak time, same for the b-wave. Independent variables were flash strength and dark adaptation time. We will consider the influence of adaptation time on amplitude first and then on peak timing.

### Amplitudes

**Fig. 2:**
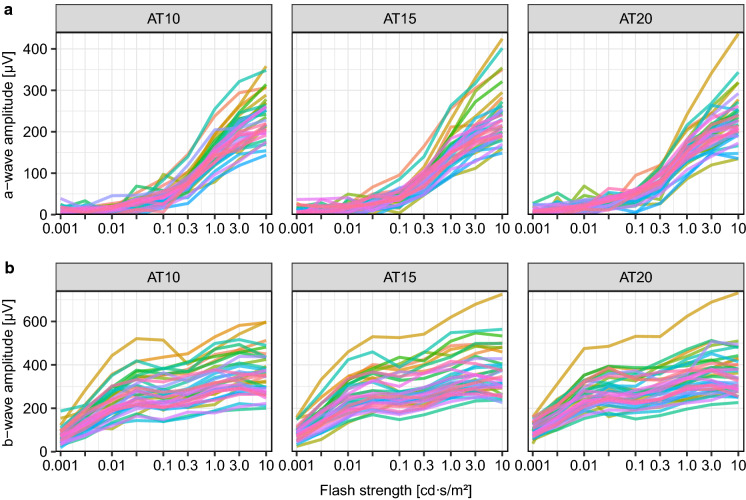
V-log-I curves (amplitude versus log(flash strength)) for all participants (indicated by color) and conditions, a-wave top, b-wave bottom. AT10, AT15 and AT20 indicate the dark adaptation times in minutes. Some outliers pop out visually, e.g., the yellowish trace at top. The a-wave shows little saturation, the b-wave more so, frequently with a “dip” at 0.1–0.3 cd s/m². From this figure, no obvious effect of dark adaptation can be recognized

Figure 2 displays the amplitudes for both a- and b-waves versus flash strength for the three DA conditions in separate panels for the a- and b-waves. The general pattern of results is consistent across the three adaptation conditions, with (1) an increase in the amplitude with flash strength for both components, (2) more saturation for the b-wave and (3) large variability across participants (more than a factor of two).

**Fig. 3:**
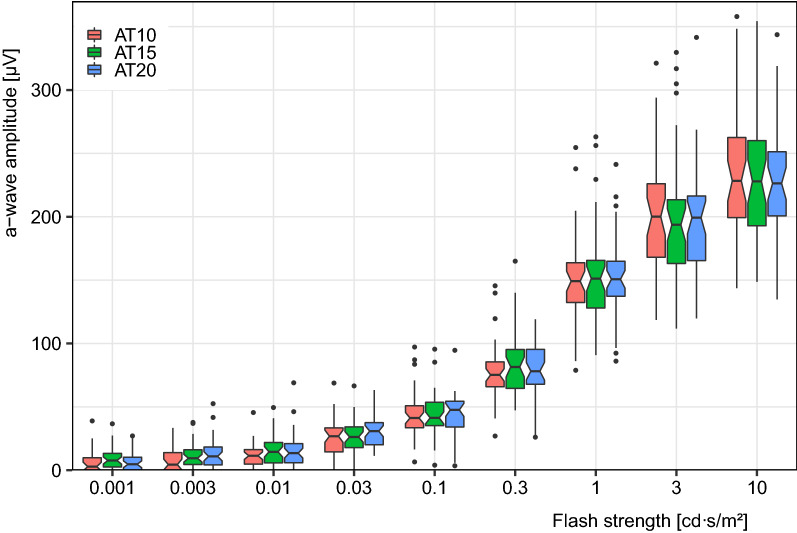
a-wave amplitude versus flash strength. For each flash strength, the three boxplots correspond to the three dark adaptation times (AT = 10, 15 and 20 min). The notches in the boxplots delineate the 95% confidence interval for the median, enabling “at-a-glance” statistical interpretation. Amplitudes increase with increasing flash strength (of course), but do not differ significantly between the dark adaptation conditions

The effect of DA duration on the a-wave amplitude can be better appreciated in the boxplots in Fig. 3. There is a hint of higher amplitudes for longer DA for weak flashes, but this is not confirmed by statistical testing.

The traces (Fig. 2, lower part) suggest that the interindividual variability is multiplicative, not additive; thus, the additive model of an ANOVA is not adequate. Consequently, we normalized the a- and b-wave amplitudes individually to the mean amplitude for the strongest flashes at AT20. The results of a two-factor ANOVA (amplitudeNormalised ~ FLASH_STRENGTH + DA_TIME) were as follows:

- a-wave: DA_TIME *p* = 0.68, FLASH_STRENGTH *p* ≪ 0.0001, interaction *p* = 0.96.
- b-wave: DA_TIME *p* ≪ 0.0001, FLASH_STRENGTH *p* ≪ 0.0001, interaction *p* = 0.0065.

**Fig. 4:**
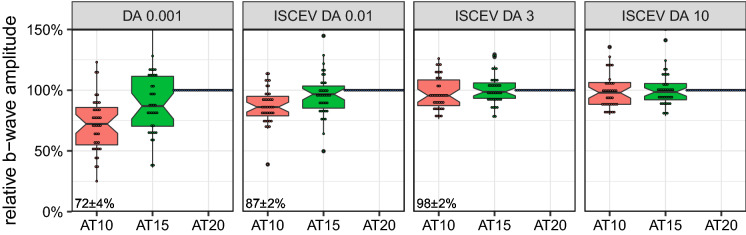
b-wave amplitude versus dark adaptation times (AT = 10, 15 and 20 min) for four different flash strengths (0.001, 0.01, 3 and 10 cd s/m²); graph arranged after ([2], their Fig. 4), individually normalized to the AT20 condition. Individual data points overlay the boxplots. For AT20 (= 100%), these form a line that may serve as a visual reference. The median percentage values for the AT10 condition are indicated at bottom left of each panel. In this rendering, the effect of DA on the b-wave amplitude becomes obvious: For AT = 10 min, the amplitude for weak flashes (0.001 cd s/m², 10 × weaker than the weakest flash from the ISCEV ERG standard) is only 72% of the one for 20 min. For the ISCEV DA 0.01, the effect is 87 ± 2%. For higher flash strengths, the effect diminishes and is no longer recognizable at ISCEV DA 3 or ISCEV DA 10

The significant effect of flash strength is trivial, of course. Given that there are no significant effects of DA duration on the normalized a-wave amplitude, Fig. 3 depicts the raw amplitudes versus flash strength for the three DA conditions. For the b-wave, in addition to flash strength (of course), the effects of DA duration are very highly significant, and there is also a highly significant interaction. Figure 4 reveals that this interaction reflects the finding that at high flash strengths (ISCEV DA 3 and DA 10) DA duration does not play a role, but for the important (rod-specific) DA 0.01 reduced DA duration does reduce amplitude, down to 87 ± 2% for AT10. The additional (non-ISCEV) DA 0.001 condition has even lower amplitude: 72 ± 4% for DA 10 relative to AT20.

### Peak times

**Fig. 5:**
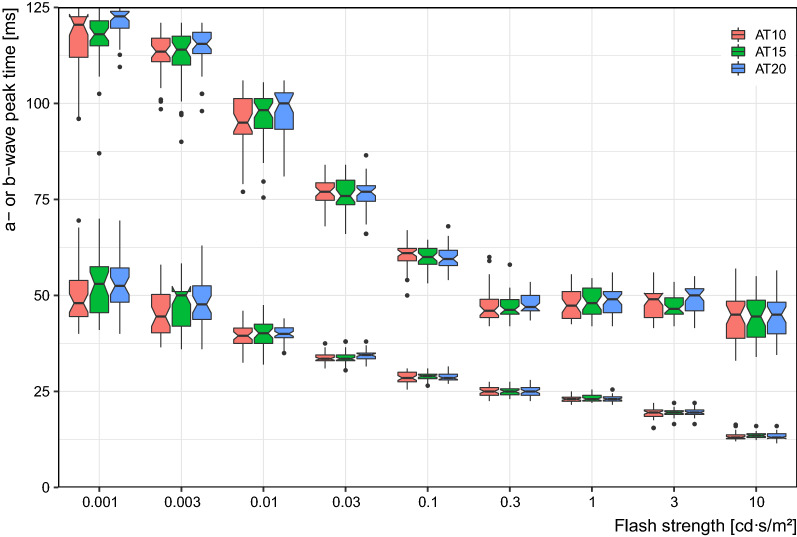
Peak times for the a-waves (lower row of data) and b-waves (upper row) versus flash strength. For each flash strength, the three boxplots correspond to the three dark adaptation times (10, 20 and 30 min). The notches in the boxplots delineate the 95% confidence interval for the median, enabling “inference by eye” statistical interpretation [8]. Peak times decrease with increasing flash strength (of course), but to not significantly differ between the dark adaptation conditions

Figure 5 displays the peak times for both a- and b-waves versus flash strength for the three DA conditions (10, 15 and 20 min). As well known, peak times decrease with increasing flash strength, borne out in this figure. There appears little effect of DA on peak times. Closer inspection shows for weak flashes slightly higher peak times for longer DA. This is borne out for the a-wave by a repeated measures ANOVA, where not only both factors were highly significant (FLASH_STRENGTH, *p* ≪ 0.0001; DA_TIME, *p* < 0.0001), but also their interaction (*p* = 0.0002). The latter represents the finding that peak times differ only for weak flashes. For the b-wave, there was the same pattern, if less significant: FLASH_STRENGTH, *p* ≪ 0.0001; DA_TIME, *p* = 0.0006; interaction, *p* = 0.015).

## Discussion

The present data suggest no effect of reducing the dark adaptation time from 20 to 10 min at high flash strengths and little (13% for DA 0.01 ERG) reduction in b-wave amplitude with weak flash strengths. This corroborates findings by Hamilton and Graham [2] who found no effect for the DA 3 ERG and a reduction of 10% for the DA 0.01 ERG when decreasing the adaptation time to 10 min. It also rhymes within error margin with Asakawa et al.’s [3] reduction to 83%: Their Table 1, “rod response,” gives 43.2 ± 10.1 µV for 20 min and 36.0 ± 7.3 µV for 10 min. Their much lower amplitudes in absolute terms are due to their use of skin electrodes.

We did not find increased intersubject amplitude variability with the 10-min adaptation, which is also consistent with the study by Hamilton and Graham [2]. Although there is a statistically significant effect of adaptation duration on peak time, it does not appear clinically relevant if one considers the interindividual variability.

The present study extends the range of flash strengths, including a nonstandard DA 0.001 ERG condition. With such very weak flashes, there was a marked effect of shortening the dark adaptation time, resulting in an 28% decrease in amplitude with 10 min of DA. This dependence of the effect of adaptation duration on flash strength may is not unexpected. On the one hand, it is intuitively plausible that an incomplete dark adaptation has most effect on the response to the weakest flash. On the other hand, for flash strengths at which cones contribute to the response, because cones reach full adaptation within 10 min [9], sensitivity to changes in dark adaptation duration is naturally reduced.

The exact effect of reducing DA time will depend on the lighting conditions before its beginning, extremes being prior photopic ERG recording (very bright), or as an opposite example, resting in a dimly lit room. Standardization of pre-dark adaptation light exposure is difficult since pupil illumination will change with every gaze change of the patient. Thus, any correction factor, if attempted, will need to account for local conditions.

Judging from the present findings, it would seem possible to reduce DA to 10 min and slightly adjust normal ranges to account for the effect found with very weak flash strengths. There is a caveat, though. The present data have been obtained in healthy young participants. It is not a given that the same results would be obtained in diseased eyes, as a number of pathological conditions are known to affect the physiological processes underlying dark adaptation [10]. Furthermore, the normal aging process is associated with changes in dark adaptation [11–13]. Finally, shortening the DA duration might increase the effect of the patient’s pre-DA exposure to different light levels.

In principle, disease-specific differences in the time course of DA can have a diagnostic value, and psychophysical dark adaptometry may be used to exploit this approach. While potentially interesting for research questions, the additional benefits of a systematic electrophysiological assessment of the complete time course in clinical routine testing by means of the ERG are not obvious, especially when taking the additional effort into account. However, as pointed out by Hamilton and Graham [2], it might be feasible to increase the diagnostic utility of the ERG by identifying a DA duration where responses show a greater difference between healthy and diseased eyes. Such an approach could possibly also achieve a better differentiation between different pathologies that yield indiscriminable ERG results with 20 min of DA.

In summary, the present data suggest that the effect of adaptation duration depends markedly on flash strength. It also confirms previous findings that the effect is relatively small within the standard ISCEV range of flash strengths with a 10-min adaptation duration.
